# Affective face biases in visual and prefrontal cortex measured with visual entrainment

**DOI:** 10.1162/IMAG.a.1206

**Published:** 2026-04-21

**Authors:** Nathan M. Petro, Yi Wei, Ilenia Salsano, Thomas W. Ward, Hannah J. Okelberry, Jason A. John, Ryan Glesinger, Lucy K. Horne, Giorgia Picci, Tony W. Wilson

**Affiliations:** Institute for Human Neuroscience, Boys Town National Research Hospital, Boys Town, NE, United States; Neuroscience and Cognitive Science Program, University of Maryland, College Park, MD, United States; Department of Pharmacology & Neuroscience, Creighton University School of Medicine, Omaha, NE, United States

**Keywords:** visual entrainment, visual competition, affective faces, magnetoencephalography, MEG

## Abstract

Facial expressions are ubiquitous and reliable social cues. Research has shown that affective faces attract attention at the cost of competing visual information, with functional neuroimaging evidence suggesting that the prefrontal cortex plays a critical role in regulating responses to emotional distractors. However, methodological constraints within neuroimaging environments often prevent the measurement of unique neural signals from multiple competing stimuli, limiting the conclusions that can be drawn regarding how affective biases in attention are generated in the brain. In the current study, we used a novel frequency tagging approach with visual entrainment during magnetoencephalography to track the unique neural signals elicited by a task-relevant Gabor patch and a concurrent, spatially overlapping face with either an angry, neutral, or happy expression. The entrainment responses were projected to the cortex using a beamformer, and a competition index was calculated per voxel to determine the bias toward either of the spatially overlapping entrained stimuli. In the prefrontal cortex, we found a stronger Gabor bias for neutral compared to angry and happy expressions, supporting prior functional neuroimaging works which point to the prefrontal cortex as critical to the regulation of emotional distractors. In the calcarine, we found a stronger face bias for angry compared to neutral and happy expressions, replicating prior findings from electroencephalography. The separate entrainment responses were also sensitive to facial expression in several regions commonly implicated in face processing, social cognition, and attention. These data highlight the utility of frequency tagging paradigms for tracking unique neural responses to concurrent and spatially overlapping stimuli, which is critical for the study of social and emotional processing.

## 1 Introduction

Facial expressions are ubiquitous and potent social cues, predictive of others’ behavior (Ekman & Friesen, 1971; Frith, 2009) and motivationally relevant environmental variables (Phelps et al., 2006). Particular expressions denoting threat or reward (e.g., angry or happy faces, respectively) are preferentially processed, often with greater speed and efficiency compared to neutral expressions (Gregory et al., 2021; Öhman, 1986; Öhman et al., 2001; Sweeny et al., 2013), and capture attention more readily than other stimuli, even in the context of competing stimuli (Pourtois, 2004; Vuilleumier, 2005). The processing of affective faces has been shown to be aberrant in most major mental health disorders (Gentili et al., 2016; MacNamara et al., 2017; Marwick & Hall, 2008; Pei et al., 2023), especially in terms of instigating exaggerated attentional biases (Abend et al., 2019; Bar-Haim et al., 2007). Thus, understanding the neural systems whereby affective faces are prioritized amid multiple stimuli is crucial to fully unpacking both normative and aberrant visuoperceptual biases.

Affective faces consistently elicit stronger activation than neutral faces across extended visual regions, including the fusiform gyrus (Fusar-Poli et al., 2009; V. I. Müller et al., 2018; Sabatinelli et al., 2011; Vuilleumier & Pourtois, 2007). These findings support the notion that affective biases in perception and attention may emerge in visual cortices, where visual information competes for neural processing resources (Desimone & Duncan, 1995; Pessoa, 2009). However, these studies largely involved the comparison of brain responses to faces presented in isolation rather than among multiple stimuli (i.e., not in competition with additional stimuli). Such an approach in neuroimaging is generally necessary to isolate neural responses to specific stimuli with a sufficient signal-to-noise ratio, but can significantly limit conclusions regarding how affective stimuli compete for attentional resources.

One method that can overcome this limitation is visual entrainment with multiple stimuli, sometimes referred to as “frequency tagging,” which involves frequency labeling individual stimuli. For example, in the simplest entrainment design, a single visual stimulus turns on-and-off at a specific frequency, thereby entraining neural populations in the visual cortex at the same frequency (Heinrichs-Graham et al., 2017a, 2017b; Norcia et al., 2015; Petro et al., 2024; Regan, 1989; Springer et al., 2022, 2023; Wiesman et al., 2019; Wiesman & Wilson, 2019). An extension of visual entrainment is frequency tagging, which involves entraining multiple concurrent stimuli at distinct frequencies. The entrained signals can be precisely distinguished from other neural signals and noise by focusing on the signal at the frequency of the entrainment using electrophysiological methods (Di Russo et al., 2007; Hillyard et al., 1997; Lopes da Silva, 1991). Studies focusing on the entrainment response have shown that attended compared to ignored (Andersen et al., 2009; Kim & Verghese, 2012; Müller et al., 2003, 2006) and emotional compared to neutral (Keil et al., 2003; Moratti et al., 2006; Petro et al., 2017) stimuli result in stronger responses in the visual cortex. Although limited, other work suggests that the strength of entrainment in the visual cortex is modulated by re-entrant feedback from fronto-parietal (Petro et al., 2017; Wieser & Keil, 2011) and temporal (Keil et al., 2009) cortices. Given the unique ability of entrainment paradigms to track cortical responses to multiple, competing stimuli (Wiesman et al., 2019; Wiesman & Wilson, 2019), frequency tagging with visual entrainment has emerged as an intriguing research tool for studying social/emotional visual processing (Wieser et al., 2016).

Early studies using frequency tagging and electroencephalography (EEG) to study emotional processing generally found that negative stimuli evoked stronger responses compared to concurrent non-emotional stimuli. Wieser et al. (2012) presented angry, neutral, or happy faces concurrently with a spatially-overlapping and task-relevant Gabor patch. The different stimuli flickered on-and-off at separate frequencies, driving two unique entrainment responses. The authors found evidence of competition between entrainment responses during angry trials, such that the face entrainment response was enhanced while entrainment toward the task-relevant Gabor was diminished. Similar emotion-driven competition effects have been shown using analogous entrainment paradigms with different types of emotional images (Bekhtereva & Müller, 2017; Deweese et al., 2016; Hindi Attar et al., 2010; Hindi Attar & Müller, 2012; Müller et al., 2008, 2011; Müller & Gundlach, 2017; Schönwald & Müller, 2014). Note that most of these previous studies focused on emotional scenes rather than faces, limiting their generalizability to face paradigms. Nonetheless, these studies suggest that emotional content (such as facial expressions) competes for representational space in the visual cortex, consistent with well-supported neurophysiological models of attention (Desimone & Duncan, 1995).

Parallel evidence from fMRI suggests that the prefrontal cortex (PFC) plays a critical role in suppressing emotional distractors, yet it remains unclear if this region influences the aforementioned competition effects in frequency tagging. In general, emotional compared to neutral stimuli elicit stronger activation along the ventral visual pathway and in the amygdala (Kober et al., 2008; Lindquist et al., 2012; Sabatinelli et al., 2011; Stevens & Hamann, 2012; Wager et al., 2015). Conversely, when presented as distractors, emotional compared to neutral stimuli usually elicit weaker right PFC responses (Dolcos et al., 2008; Dolcos & McCarthy, 2006; Marxen et al., 2021; Oei et al., 2012; Padmala & Pessoa, 2011; Perlstein et al., 2002). Together, these findings suggest that distracting emotional content receives prioritized “bottom-up” processing vis-à-vis ventral cortical pathways, while PFC regions may diminish this effect (Dolcos et al., 2006) insofar as they direct dorsal attention processes toward behavioral goals (Dolcos et al., 2013; Iordan et al., 2013; Padmala & Pessoa, 2011; Pessoa, 2017; Shafer & Dolcos, 2012). However, as mentioned above, this conclusion is speculative given that the timing of these responses relative to one another is unknown and would significantly impact the interpretation. Along these same lines, earlier studies using temporally resolved methods like EEG were conducted at the electrode-level and thus were unable to identify the precise spatial origins of entrainment effects. Thus, whether the PFC is central to such processes remains poorly understood.

In the current study, we used a dynamic functional mapping approach based on high-density magnetoencephalography (MEG) to quantify simultaneous frequency tagged entrainment responses, and then computed a competition index that reflects the differential entrainment strength (i.e., bias) toward a Gabor patch or an affective facial expression. Briefly, we leveraged a design first proposed by (Wieser & Keil, 2011; Wieser et al., 2012) in which separate visual entrainment “tags” were used for the task-relevant stimulus (i.e., a Gabor patch) and a concurrent, spatially overlapping, task-irrelevant angry, neutral, or happy facial expression. The competition indices (i.e., Gabor versus each facial expression) were compared among affective and neutral expressions using a whole-brain, voxel-wise repeated measures ANOVA. We predicted that the entrainment responses would be biased toward faces for affective compared to neutral expressions in the visual cortex, whereas entrainment responses would be biased toward the competing Gabor patch for the non-emotional neutral faces in dorsal attention cortices. Beyond the competition index, we also compared entrainment responses directly as a function of facial expression. Here, we predicted that entrainment to the faces would be stronger for the affective relative to neutral faces in the ventral visual and temporal cortex, consistent with meta-analyses of affective face processing (Fusar-Poli et al., 2009; Müller et al., 2018; Sabatinelli et al., 2011). Finally, we predicted weaker Gabor entrainment in fronto-parietal attention cortices during trials with affective compared to neutral facial expressions.

## 2 Methods

### 2.1 Participants

A total of 34 adults (20 females) with a mean age of 31.21 (SD = 7.24) years were included in this study. One participant was left-handed. Exclusionary criteria included any medical illness affecting CNS function (e.g., HIV/AIDS, Lupus, etc.), any neurological or psychiatric disorder, cognitive impairment, history of head trauma, current substance misuse, and the standard exclusionary criteria related to MEG and MRI acquisition (e.g., ferromagnetic implants). The Institutional Review Board at Boys Town National Research Hospital reviewed and approved this investigation. Each participant provided written informed consent following a detailed description of the study.

### 2.2 Experimental paradigm

During the MEG recording, participants sat in a nonmagnetic chair within a magnetically shielded room. All stimuli were presented using the Psychophysics Toolbox (Kleiner et al., 2007) and a PROPixx DLP LED projector (VPixx Technologies Inc., Saint-Bruno-de-Montarville, Canada) with a refresh rate of 480 Hz. The experiment consisted of 264 trials. In each trial, a face was presented centrally for 2000 ms, during which time the face flickered on-and-off at a rate of 34.3 Hz. A Gabor patch with 41% transparency was presented concurrently and superimposed on top of the face; it flickered on-and-off at a rate of 18.5 Hz. The Gabor patch contrasted from gray to white and was presented at a 45-degree angle. The duty cycle of the flicker streams was 50%. These two frequencies were selected to avoid possible contamination between either fundamental frequency and their (sub)harmonics, and to avoid the alpha-band which is known to show strong desynchronization during visual stimulus presentation.

**Fig. 1.**
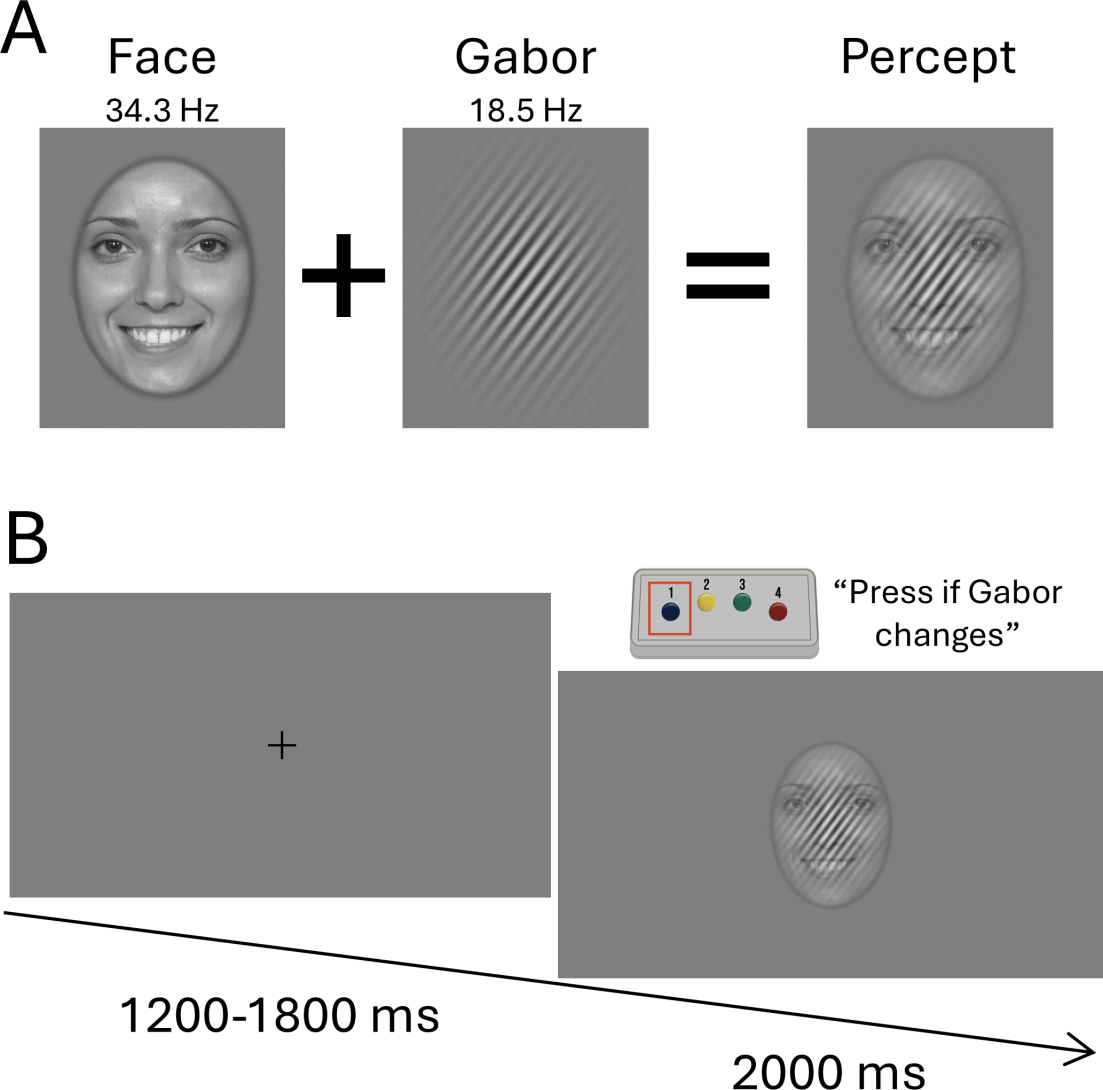
Stimuli and Task Design. (A) The stimuli consisted of a face with a concurrently presented and superimposed Gabor patch, which were flickered on-and-off at 34.3 and 18.5 Hz, respectively. (B) Each trial consisted of a fixation cross for a duration of 1200-1800 ms, and the face-Gabor percept for 2000 ms. Participants were instructed to attend to the Gabor patch and respond via button pad if the Gabor changed from a 45- to 0-degree angle during the trial, which occurred on 11% of all trials. These oddball trials were not included in the final analyses.

On 24 of the 264 trials, the Gabor patch changed from a 45-degree angle (i.e., tilted to the right) to a 0-degree angle (i.e., horizontally flat) at a random interval between 500–1375 ms following flicker onset. Participants were instructed to press a button with their index finger when they detected this change. These 24 “oddball” trials, as well as any standard trials with a false-alarm response, were discarded from subsequent analyses. Preceding each trial, a fixation cross was presented centrally for a duration between 1200 and 1800 ms (Fig. 1). All stimuli were presented on a gray background.

The face stimuli were 264 images with an angry, neutral, or happy expression drawn from the RADIATE (Conley et al., 2018), Chicago (Ma et al., 2015), Umeå (Samuelsson et al., 2012), London (DeBruine & Jones, 2017), and KDEF (Lundqvist et al., 1998) databases. Male and female actors were equated within each expression condition. Images were cropped into ovals with feathered edges to remove external features (e.g., ears and the majority of hair) and were converted to black and white. Lastly, the overall luminance of each image was equated using the SHINE toolbox (Willenbockel et al., 2010) in MATLAB. Stimuli were presented in a pseudo-randomized order such that the same sex or affective expression condition were repeated in no more than two consecutive trials.

### 2.3 MEG and MRI data acquisition

All MEG recordings were conducted in a two-layer magnetically-shielded VACOSHIELD room (Vacuumschmelze, Hanau, Germany). Neuromagnetic responses were sampled continuously at 1 kHz, with an acquisition bandwidth of 0.1–330 Hz, using a MEGIN Triux Neo MEG system with 306 magnetic sensors (Helsinki, Finland). During data acquisition, participants were monitored via real-time audio-visual feeds from inside the shielded room. Participant-wise MEG data were corrected for head motion and subjected to external noise reduction using signal space separation method with a temporal extension (Taulu & Simola, 2006). High-resolution structural images were collected using a 1-mm isotropic MPRAGE sequence [TR = 2.4 s, TE = 2.05 ms, flip angle = 8°, FOV = 256 mm] on a Siemens Prisma 3T scanner with a 32-channel head coil.

### 2.4 Structural MRI processing, and MEG coregistration

Preceding MEG measurement, five head position indicator (HPI) coils were attached to the participant’s head and localized, together with three fiducial points and at least 100 scalp surface points, with a 3D digitizer (Fastrak, Polhemus Navigator Sciences, Colchester, VT, USA). Once in the MEG, electrical currents with unique frequency labels (e.g., 322 Hz) were fed into each of the HPI coils, which induced measurable magnetic fields, allowing the position of the coils to be actively tracked relative to the MEG sensors throughout the recording. Since the HPI coil locations were also known in head coordinates, all MEG measurements could be transformed into a common coordinate system. With this coordinate system, participant-wise MEG data were coregistered with the participant’s high-resolution structural T1-weighted MRI data prior to source reconstruction using BESA MRI (Version 3.0, BESA GmbH, Gräfelfing, Germany). Structural MRI data were transformed into standardized space and aligned parallel to the anterior and posterior commissures. Following source analysis, each participant’s MEG functional images were also transformed into standardized space and spatially resampled to enable comparison across participants.

### 2.5 MEG preprocessing, time-frequency transformation, and sensor-level statistics

Cardiac and ocular artifacts (blinks and eye movements) were removed from the data using signal-space projection (SSP), which was accounted for during source analysis (Uusitalo & Ilmoniemi, 1997). The continuous magnetic time series was divided into 2800 ms epochs (-600 to 2200 ms surrounding stimulus onset), with the baseline period being defined as the 600 ms prior to the onset of the face/Gabor flicker stream (i.e., -600 to 0 ms). Subsequently, epochs with remaining artifacts were removed based on a fixed threshold method, supplemented with visual inspection. Briefly, the amplitude and gradient distributions across all trials were determined per participant, and those trials containing the highest amplitude and/or gradient values relative to this distribution were rejected based on participant-specific thresholds. This approach was employed to minimize the impact of individual differences in sensor proximity to the brain and overall head size, which strongly affects MEG signal amplitude.

To identify the precise temporal and spectral extent of the entrainment responses, the artifact-free epochs were transformed into the time-frequency domain using complex demodulation (Hoechstetter et al., 2004; Kovach & Gander, 2016; Papp & Ktonas, 1977), with a resolution of 0.25 Hz and 200 ms between 4 and 40 Hz. Following time-frequency transformation, spectral power estimates per sensor were averaged across trials to generate plots of mean spectral density per sensor. These sensor-level data were then normalized to the baseline power within each frequency bin, which was calculated as the mean power for that 0.25 Hz bin during the -600 to 0 ms time period. The significant time-frequency windows used for source imaging were then determined by statistical analysis of the sensor-level spectrograms across all participants, conditions, and the entire array of 204 gradiometers using BESA Statistics (Version 2.1T, BESA GmbH, Gräfelfing, Germany). Briefly, each pixel per spectrogram was initially evaluated using a mass univariate approach based on the general linear model, followed by cluster-based permutation testing to address the problem of multiple comparisons (Ernst, 2004; Maris & Oostenveld, 2007). This two-stage procedure was utilized to minimize false positive results while maintaining sensitivity, with the first stage consisting of paired-sample *t*-tests against baseline on each pixel per spectrogram and thresholding the output spectrograms of *t*-values at *p* < .05 to define time-frequency bins containing potentially significant deviations from baseline. In stage two, the time-frequency bins that survived thresholding (at *p* < .05) were clustered with temporally and/or spectrally neighboring bins that also survived, and cluster values were derived by summing all *t*-values within each cluster. Nonparametric permutation testing was then used to derive a distribution of cluster-values, and the significance level of the cluster(s) was tested directly using this permuted distribution, which was the result of 10,000 permutations. Based on this cluster-based permutation analysis, only the time-frequency windows that contained significant deviations from baseline at the *p* < .001, corrected, threshold across all participants and conditions were subjected to source imaging (i.e., beamforming).

### 2.6 MEG source imaging and statistics

Neural responses were imaged through a time-frequency-resolved extension of the linearly constrained minimum variance (LCMV) beamformer (Dalal et al., 2006; Gross et al., 2001; Van Veen et al., 1997). The images were derived from the cross spectral densities of all combinations of MEG gradiometers averaged over the time-frequency range of interest, and the solution of the forward problem for each location on a 4 x 4 x 4 mm grid specified by input voxel space. In principle, the beamformer operator generates a spatial filter for each grid point that passes signals without attenuation from a given neural region, while suppressing activity in all other brain areas. The filter properties arise from the forward solution (i.e., lead field matrix) for each location on a volumetric grid specified by input voxel space, and from the MEG cross spectral density matrix. Basically, for each voxel, a set of beamformer weights is determined, which amounts to each MEG sensor being allocated a sensitivity weighting for activity in that particular voxel. Following convention, the source power in these images was normalized per participant using a pre-stimulus period (i.e., baseline) of equal duration and bandwidth (Hillebrand et al., 2005). Such images are typically referred to as pseudo-*t* maps, with units (pseudo-*t*) that reflect noise-normalized power differences (i.e., active vs. passive) per voxel. MEG pre-processing and imaging used the Brain Electrical Source Analysis (version 7.1) software. Individual participant-level maps containing significant artifacts were excluded from further analysis.

### 2.7 Competition index of the entrainment responses

We derived a competition index to assess differences in neural entrainment between the two flickering stimuli (i.e., differences in the relative entrainment between the face and concurrent Gabor patch). This competition index was calculated similar to Wieser et al. (2012), which represents the ratio of face versus Gabor entrainment, separately for each voxel. Briefly, to compare the face and Gabor entrainment signals, the oscillatory activity at each driving frequency was separately normalized (i.e., z-transformed) per voxel across the three expression conditions. In other words, the Gabor entrainment responses for each of the three expression conditions were transformed to a z-scale using a distribution that included the Gabor responses across all three facial expression conditions. Separately, the face entrainment responses for each of the three expression conditions were transformed to a z-scale using a distribution that included all three facial expression conditions. These normalized z-scores were made positive by adding constant of 5, in order to simplify the interpretation. This normalization procedure allowed the strength of the entrainment responses to be directly compared to each other, given that they are known to naturally vary in amplitude across the spectrum (Herrmann, 2001; Lazarev et al., 2001; Pastor et al., 2003, 2007; Petro et al., 2024).

Subsequently, we computed the ratio between face and Gabor entrainment per expression condition using these normalized values (i.e., face/face + Gabor entrainment). For clarity and ease of interpretation, we expressed this ratio as a percentage of the denominator. Thus, a ratio above 50% indicates an entrainment bias toward the face, whereas a ratio below 50% indicates an entrainment bias toward the task-relevant Gabor. These competition indices were computed separately at each voxel, resulting in a whole brain map of competition between the face and Gabor entrainment responses for each expression condition.

### 2.8 Whole-brain statistics

To identify differences in the competition indices as a function of facial expression, voxel-wise whole-brain ANOVAs with expression (angry, neutral, happy) as the within-subjects factor were computed in *R* using the competition index maps. To account for multiple comparisons, a cluster forming threshold of *p* < .005 and cluster-extent threshold of *k* > 10 (i.e., at least 640 mm^3^ of brain tissue) were used, based on Gaussian random fields theory (Poline et al., 1995; Worsley et al., 1996). To determine the direction of the effect for any significant cluster, follow-up paired *t*-tests were conducted on the voxels showing the peak-effect (i.e., the voxel with the highest statistical value per cluster). Finally, the same ANOVA approach was conducted on the functional maps representing the face and Gabor entrainment responses separately to determine the effect of facial expression. Note that these analyses used the pseudo-t voxel values from the beamformer and not the normalized z-scale values since the comparisons were between entrainment responses at the same frequency.

## 3 Results

Participants performed well on the oddball task, with a mean accuracy rate of 92.64% (SD = 11.19%). Following artifact rejection and exclusion of incorrect trials, each participant had an average of 75.44 (SD = 3.69), 75.85 (SD = 2.77), and 75.30 (SD = 3.66) trials for angry, neutral, and happy conditions, respectively. The number of retained trials did not differ across facial expression conditions (*F*_2,66_ = 1.67, *p* > .05).

### 3.1 Sensor-level neural responses

**Fig. 2.**
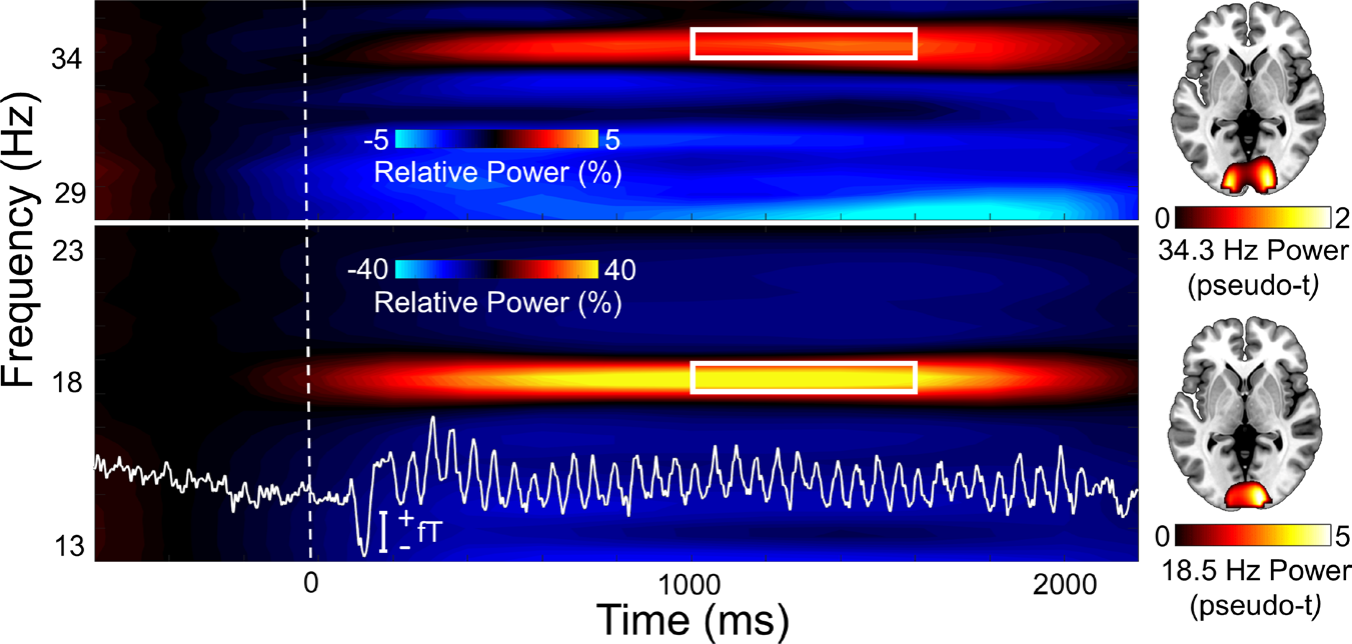
Sensor- and source-level entrainment responses. (Left) Time-frequency spectrograms illustrate the responses across all trials and participants from a representative sensor (MEG1713). Time (ms) is shown on the x-axis and frequency (Hz) on the y-axis, and the color scale illustrates the change in power relative to the baseline period. Strong increases at each of the entrainment frequencies (18.5 and 34.3 Hz) were observed. The time series for one representative participant and sensor is drawn in white. White boxes designate the time-frequency windows submitted to beamformer imaging. (Right) Mean beamformer functional maps (pseudo-*t*; see color bar) for each entrainment response (computed based on the white boxes) collapsed across all participants and the three facial expression conditions.

To derive the time-frequency bins for beamforming analyses, sensor-level spectrograms were probed using non-parametric permutation testing (see section 2.5). This revealed significant responses in a large number of posterior sensors for both the Gabor and face entrainment frequencies (18.5 Hz and 34.3 Hz, respectively). We focused on 1 Hz wide bands surrounding each entrainment frequency and the time window with the strongest response amplitude, which was 1000-1600 ms following flicker onset (*p* < .001, corrected; Fig. 2). These time-frequency windows were imaged using baselines of equal bandwidth and duration (0 to -600 ms). Note that we did not image any harmonic responses. Following imaging, each entrainment response was grand-averaged across all participants and conditions to assess data quality and to visualize the brain regions generating the strongest responses.

### 3.2 Functional mapping of competition indices as a function of expression

**Fig. 3.**
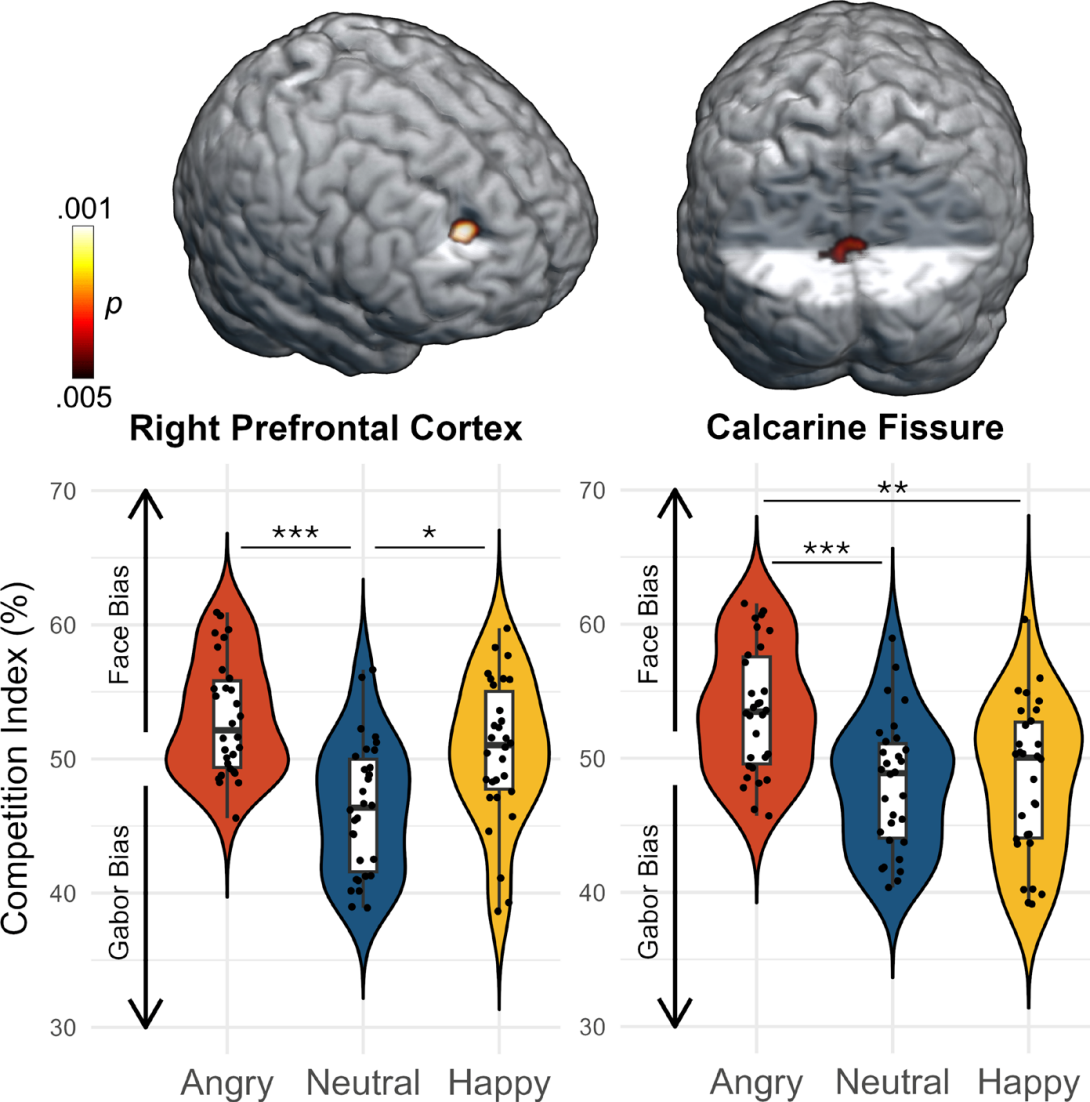
Whole-brain differences in the entrainment competition indices between trials with angry, neutral, and happy faces. The brain images illustrate the *p*-values for the effect of facial expression thresholded at *p* < .005, corrected. In the data plots, the dots represent the competition index for each participant, taken from the peak voxel in the corresponding clusters. Values over 50% reflect a bias toward the face, whereas values below 50% indicate a bias toward the Gabor. The box plots illustrate the mean, first, and third quartiles, and the whiskers indicate the minima and maxima. The violin plots illustrate the probability density. Horizontal bars depict the significant pairwise differences (***p < .001, **p < .01, *p < .05).

Competition between the two entrainment responses (i.e., the competition index) showed an effect of expression in two clusters, located in the right prefrontal cortex (PFC; *F*_2,58_ = 9.77, *p* < .001, corrected) and in the calcarine fissure (*F*_2,58_ = 7.30, *p* < .005, corrected; Fig. 3). Follow-up paired *t*-tests revealed a lower competition index (i.e., bias toward the Gabor) in the right PFC during neutral relative to angry (*t*_29_ = -4.86, *p* < .001) and happy (*t*_29_ = -2.53, *p* < .05) trials, while angry and happy trials did not differ (*t*_29_ = -1.67, *p* > .05). In the calcarine fissure, follow-up paired *t*-tests revealed a bias toward the face (i.e., a higher competition index) for angry relative to neutral (*t*_29_ = 3.93, *p* < .001) and happy (*t*_29_ = 3.06, *p* < .01) trials, while neutral and happy trials did not differ (*t*_29_ = 0.01, *p* > .05).

### 3.3 Functional mapping of separate entrainment responses as a function of expression

**Fig. 4.**
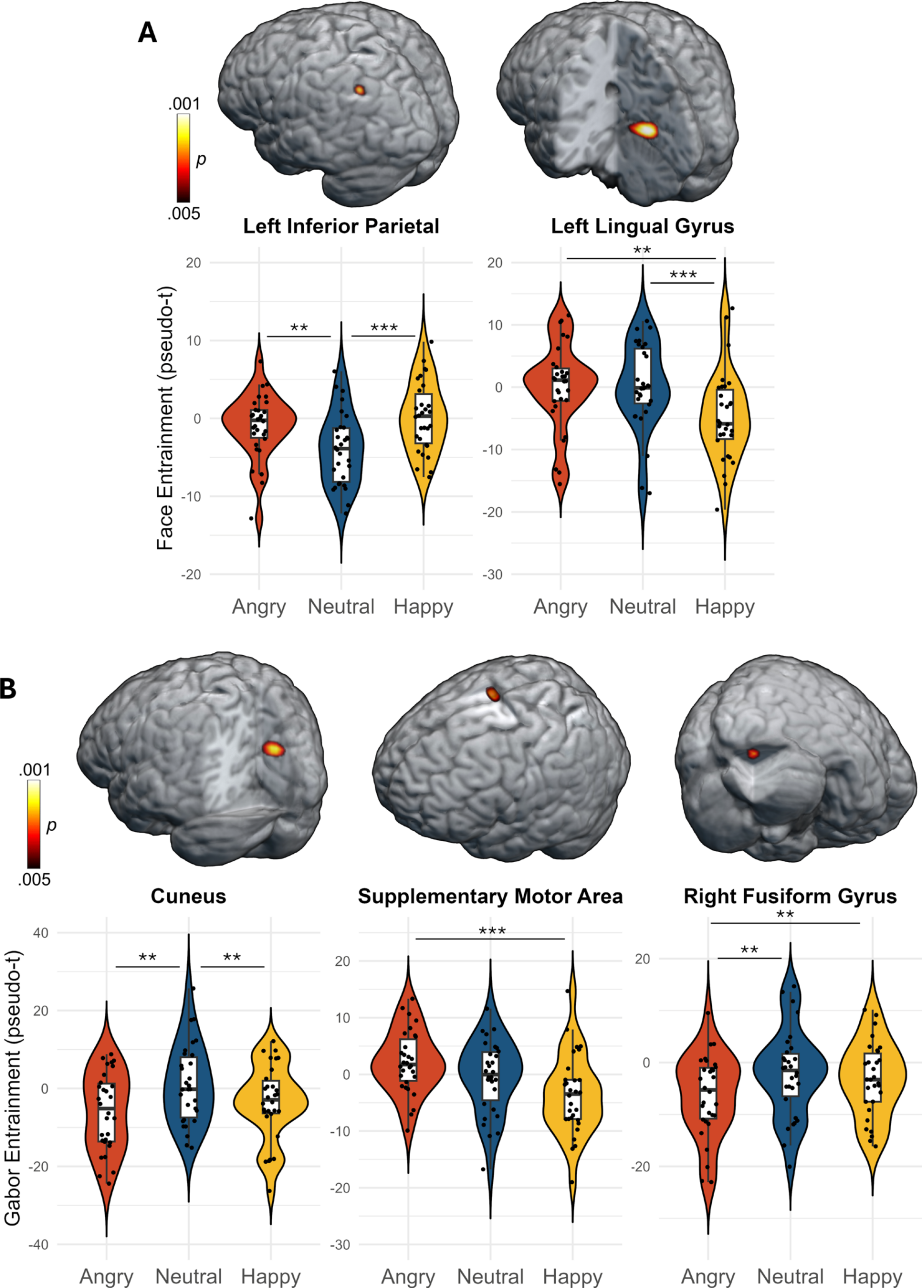
Whole-brain ANOVA maps of the entrainment response differences between trials with angry, neutral, and happy faces for the (A) face and (B) Gabor entrainment responses. In each panel, the brain images illustrate the *p*-values from the effect of facial expression thresholded at *p* < .005, corrected. In the data plots, the dots represent the entrainment strength for each participant per condition, taken from the peak-voxel in the corresponding clusters. The box plots show the mean, first and third quartiles, and the whiskers indicate the minima and maxima. The violin plots illustrate the probability density. Horizontal bars depict the significant pairwise differences (***p < .001, **p < .01).

An ANOVA focusing on the entrainment response to the faces showed an effect of expression in two clusters, located in the left lingual gyrus (*F*_2,58_ = 8.18, *p* < .001, corrected) and the left inferior parietal cortex (*F*_2,58_ = 8.00, *p* < .001, corrected; Fig. 4). For the left lingual gyrus, entrainment was weaker for happy compared to angry (*t*_29_ = -3.18, *p* < .01) and neutral faces (*t*_29_ = -4.16, *p* < .001), but did not differ between angry and neutral faces (*t*_29_ = 0.02, *p* > .05). For the left inferior parietal, entrainment was weaker for neutral compared to angry (*t*_29_ = 2.83, *p* < .01) and happy faces (*t*_29_ = -3.81, *p* < .001), but did not differ between angry and happy faces (*t*_29_ = 1.07, *p* > .05).

Entrainment to the task-relevant Gabor patch showed an effect of expression in three clusters, located in a medial portion of the supplementary motor area (*F*_2,58_ = 8.01, *p* < .001, corrected), a medial portion of the cuneus (*F*_2,58_ = 7.77, *p* < .005, corrected), and the right fusiform gyrus (*F*_2,58_ = 7.27, *p* < .005, corrected; Fig. 4). For the supplementary motor area, follow-up tests indicated that Gabor entrainment was stronger during angry compared to happy faces (*t*_29_ = 5.09, *p* < .001). There were also trends for Gabor entrainment during neutral faces to be weaker than angry (*t*_29_ = -1.82, *p* = .079) and stronger than happy faces (*t*_29_ = 1.85, *p* = .074). For the cuneus, Gabor entrainment was stronger during neutral compared to angry (*t*_29_ = 3.24, *p* < .01) and happy faces (*t*_29_ = 3.09, *p* < .01), while Gabor entrainment did not differ during angry and happy faces (*t*_29_ = 1.47, *p* > .05). For the fusiform gyrus, Gabor entrainment was weaker during angry faces compared to neutral (*t*_29_ = -3.11, *p* < .01) and happy faces (*t*_29_ = -3.37, *p* < .01), while neutral and happy faces did not differ (*t*_29_ = 1.03, *p* > .05).

## 4 Discussion

In the current study, we used frequency tagging to track the unique neural responses to a task-relevant Gabor patch and a concurrent, spatially-overlapping, task-irrelevant facial expression. To assess the degree to which different facial expressions biased neural population responses toward the face or the task-relevant Gabor patch, we computed a competition index using the unique entrainment signature of each stimulus type. We found a stronger bias toward faces in the calcarine fissure in the angry compared to the happy and neutral conditions, and a stronger bias toward the Gabor patch in the prefrontal cortex during neutral compared to angry and happy expressions. In addition, the separate entrainment responses were sensitive to expression in several visual and attention regions in ventral visual and parietal cortices, as well as the supplementary motor area. The implications of these findings are discussed below.

The competition effect in the calcarine fissure showed that during angry face trials, there was a stronger face bias over the Gabor patch compared to trials with happy or neutral faces. This pattern closely resembles that observed in Wieser et al. (2012), suggesting that their electrode-level results were likely driven by low-level visual processes near or within the calcarine. Interestingly, this effect was observed in those with relatively high social anxiety (Wieser et al., 2012), whereas we observed it across all participants in the current study. This difference may be due to the use of source imaging, which enables the contribution of specific regions (e.g., visual cortex) to be isolated from that of other regions such as the PFC, where neutral and happy faces showed a different pattern of effects in the current study. Regardless, these findings indicate important effects of emotional expression in early visual cortices.

The competition index also differed by facial expression in the right PFC, pointing to its role in coordinating attention amid emotional distraction. Specifically, the PFC has been consistently implicated in regulating attention by flexibly allocating cognitive resources and suppressing emotionally salient, task-irrelevant stimuli (Anticevic et al., 2010; Dolcos et al., 2008, 2013; Dolcos & McCarthy, 2006; Iordan et al., 2013; Marxen et al., 2021; Oei et al., 2012; Padmala & Pessoa, 2011; Perlstein et al., 2002; Pessoa, 2017; Shafer & Dolcos, 2012). In agreement with our results, such emotional effects in the PFC tended to be right-lateralized in these prior works. Beyond its role in modulating emotional distraction, the PFC is active during cognitive interference tasks (Son, Arif, et al., 2024; Son, Killanin, et al., 2024) and during working memory tasks that involve distractors during the maintenance phase (Hall et al., 2024). Within this framework, the shift toward stronger engagement of face-related signals during angry and happy trials may reflect an increased role in managing attentional control under conditions of increased emotional salience, regardless of whether the stimulus is task relevant.

Along a similar vein, activation of the PFC is consistently observed during explicit emotional regulation tasks (Messina et al., 2021; Morawetz & Basten, 2024; Petro et al., 2018). Here, regions of the PFC are thought to communicate with the amygdala to perhaps inhibit emotion-related activation (Gee et al., 2013; Ochsner et al., 2012; Urry, 2006) and/or reciprocally strengthen attention toward emotional information coded in the amygdala (Pessoa, 2017). The overall pattern of competition effects in the PFC suggests that these findings may reflect emotion regulation mechanisms and highlight the unique capacity of frequency tagging paradigms to illuminate specific neural responses to competing features in complex visual environments. Indeed our novel entrainment paradigm provides evidence to support the role of the PFC in regulation, to the extent that it is involved in distinguishing the emotional salience of competing visual content present simultaneously with spatial overlap. This constitutes an important extension of PFC findings from functional MRI (fMRI), as the sluggish temporal resolution of the hemodynamic response necessitates that task-relevant stimuli be temporally separate from emotional distractors in order to identify their unique impacts. In this regard, the current results support fMRI findings indicating that the PFC has an important role in processing emotional stimuli amid limited attention resources (Dolcos et al., 2020; Pessoa, 2017).

Beyond the competition indices, we quantified the individual entrainment responses separately per expression condition. Entrainment to the faces was weakest for neutral compared to emotional faces in the inferior parietal cortices. The inferior parietal is known to be a key region in the guidance of attention (Corbetta & Shulman, 2002), integrating sensory information with behavioral goals (Kastner et al., 2017; Meyer et al., 2025) and is active across a broad array of cognitive tasks (Foster et al., 2022; Igelström & Graziano, 2017). The stronger entrainment for emotional compared to neutral faces thus indicates that distracting, task-irrelevant emotional expressions more strongly engage cognitive processes in this region and perhaps attract attention in a bottom-up fashion. In addition, within the lingual gyrus, entrainment was stronger to angry and neutral compared to happy faces. The lingual gyrus, containing regions V1-V4, performs many basic visual functions (Fung et al., 2021) and has been implicated in processing facial features (Benuzzi et al., 2007; Puce et al., 1995), especially emotional expressions (Fusar-Poli et al., 2009; Iidaka et al., 2002; Kilts et al., 2003; Nomi et al., 2008; Sato et al., 2004). Previous studies have also implicated this area in visual memory (Bogousslavsky et al., 1987; Kondo et al., 2005; Zhang et al., 2019, 2016), imagery (D’Esposito et al., 1997; Kim et al., 2007; Olivetti Belardinelli et al., 2009), and semantic processing (Zhang et al., 2014). Thus, stronger entrainment to facial stimuli and differences based on emotional expressions clearly align with the known functions of these areas.

A collection of regions also exhibited stronger entrainment to the task-relevant Gabor patch, which varied based on the superimposed emotional expression. In the anterior cuneus, entrainment to the Gabor was weaker during emotional compared to neutral faces, whereas in the fusiform Gabor entrainment was weakest for angry compared to neutral and happy trials. In the supplementary motor area, Gabor entrainment was stronger during angry relative to happy trials and neutral faces did not differ from either of the emotion faces. The cuneus (Arroyo et al., 1997; Knight et al., 2024; Lee et al., 2015; Vanni et al., 2001) is known to be involved in early, low-level visual processing and possess connections with striate and extra-striate visual cortex (Palejwala et al., 2021). The cuneus also is involved in guiding attention using predictive visual cues (Burgess et al., 2001, 2003) and goal-directed saccades (O’Driscoll et al., 2000). Broadly, the fusiform region integrates visual information to form representations (Blauch et al., 2022; Scott & Arcaro, 2023) and has long been viewed as a critical node in face processing (Fusar-Poli et al., 2009; Haxby et al., 2000, 2002; Kanwisher & Yovel, 2006; Müller et al., 2018). Moreover, the fusiform is known to exhibit stronger activation during processing of emotional faces (Allison et al., 2000; Müller et al., 2018; Puce et al., 1998; Sabatinelli et al., 2011). Perhaps surprisingly, the fusiform gyrus effect was observed for Gabor, rather than face entrainment, but, nonetheless, these results suggest that negative facial expressions affect processing of other stimuli in these regions. Regarding the supplementary motor area, our results may indicate that engagement with the task-relevant stimulus (which requires a motor-decision) is impacted by the presence of concurrent angry faces, consistent with similar studies showing diminished task accuracy with competing emotional stimuli (Bekhtereva & Müller, 2017; Hindi Attar et al., 2010; Hindi Attar & Müller, 2012; Müller et al., 2008, 2011; Schönwald & Müller, 2014). Further, previous imaging studies have also found stronger (pre-) SMA activation for emotional compared to neutral faces and scenes (Kober et al., 2008; Müller et al., 2018; Petro, Livermore, et al., 2025; Petro, Schantell, et al., 2025; Sabatinelli et al., 2011). This finding enriches the notion that activation of motor systems is a fundamental aspect of emotion processing (Frijda, 2000; Lang & Bradley, 2010), but further work is needed.

Before closing, it is important to acknowledge the limitations of this study. First, behavioral responses were made only during oddball trials in order to minimize contamination of the entrainment response with motor-related brain activity. Future work should consider adapting this paradigm to examine how behavioral responses are related to either entrainment response, although entrainment frequencies should be carefully selected to avoid the well-known beta responses seen in the motor cortex during movement (Heinrichs-Graham et al., 2018; Heinrichs-Graham & Wilson, 2016; Wilson et al., 2011, 2016). In addition, some work has found that visual regions “prefer” particular entrainment frequencies (Herrmann, 2001; Lazarev et al., 2001; Pastor et al., 2003, 2007; Petro et al., 2024). Along these lines, different frequencies should be tested to determine whether the current results generalize to other driving frequencies outside of 18.5 and 34.3 Hz. In this regard, it is possible that particular driving frequencies may “resonate” with the activity in specific brain regions (Canolty et al., 2010; Pastor et al., 2003), and perhaps influence different aspects of cognition and perception. Lastly, the current sample consisted of healthy adults; future studies should test if these findings extend to younger and older participants and examine patient populations where changes in affective face processing have been reported (Gentili et al., 2016; MacNamara et al., 2017; Marwick & Hall, 2008; Pei et al., 2023).

To summarize, we used frequency tagging to entrain and quantify neural responses to individual stimuli that were spatially overlapping and presented simultaneously, with a Gabor patch being the task relevant stimulus and facial expressions being the distracting task-irrelevant stimuli. We found that entrainment responses in the calcarine were more strongly biased toward the face for angry compared to neutral and happy expressions, consistent with prior findings from EEG. This finding also suggests that perceptual biases toward emotional faces involve a bias in early visual circuitry, consistent with popular neurophysiological models of attention (Desimone & Duncan, 1995; Pessoa, 2009) and the notion that facial features are rapidly extracted in perception to aid in the efficient interpretation of the expression’s emotion and meaning (Adolphs, 2002; Haxby et al., 2000). In contrast, neural responses in the right PFC were biased more strongly toward the Gabor during neutral face trials relative to angry and happy trials, possibly reflecting this region’s role in regulating responses to distracting emotional information. Lastly, examining the entrainment responses separately revealed brain regions that were sensitive to facial expression differences in several attention and face processing regions, as well as in the supplementary motor area. Together these results highlight the utility of using visual entrainment to “tag” individual stimuli and thereby track the neural responses unique to spatially overlapping stimuli within complex displays. We envision that this approach may be highly valuable in the context of social and emotional processing, where emotionally charged content may attract attention away from other behaviorally relevant stimuli.

## Data Availability

The data used in this article will be made publicly available through the COINS framework at the completion of the study (https://coins.trendscenter.org/). Code may be made available upon reasonable request.
